# Supplementary figures and images for: Comparing fNIRS signal qualities between approaches with and without short channels
Source: PLoS One. 2020 Dec 23;15(12):e0244186. doi: 10.1371/journal.pone.0244186 (PMC7757903; doi:10.1371/journal.pone.0244186)

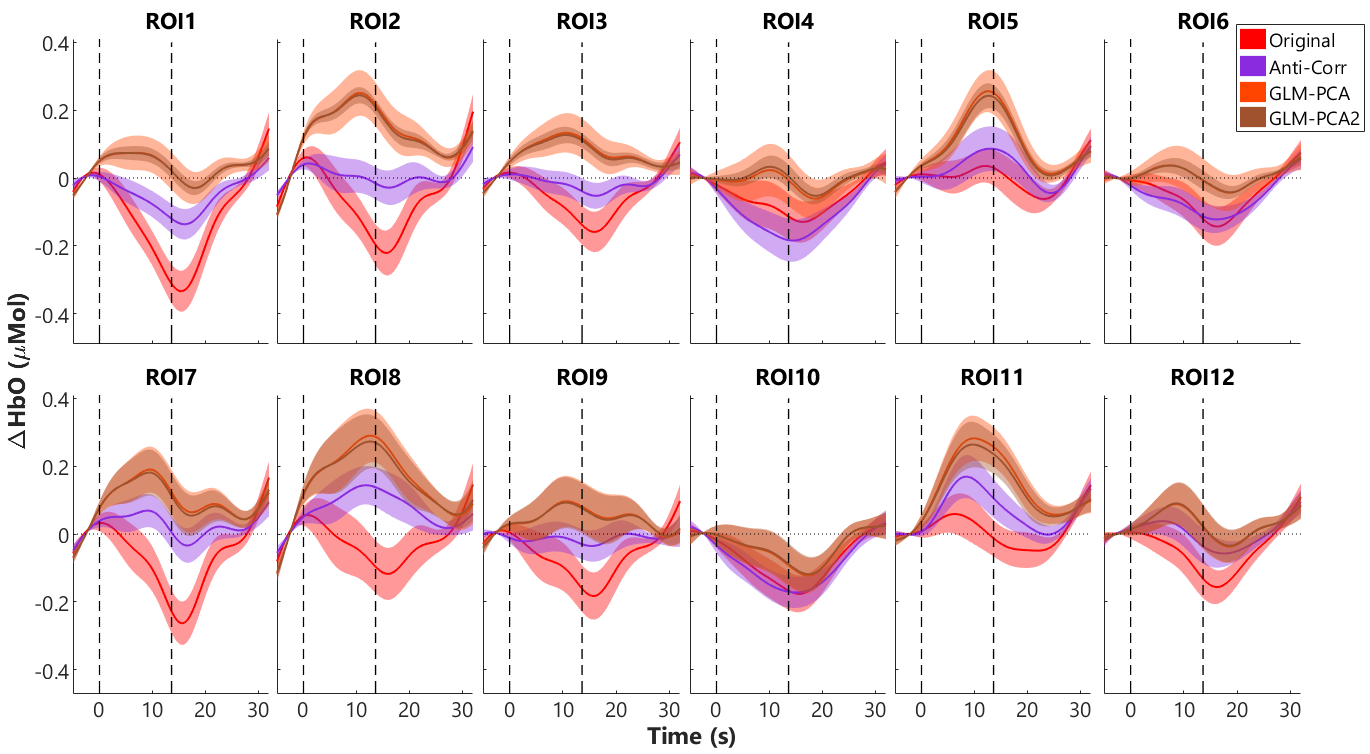

Supplement: S1 Fig — Results in channels with SCI> = 0.75 were included. The block-average results, i.e., group means (solid lines) and standard error of means (SEM, shaded areas) of ΔHbO responses without further reducing the systemic responses (Original, red) and after applying the Anti-Corr (purple), the GLM-PCA (with stimulus-related regressors and 2 PCs, yellow), and the GLM-PCA2 (with the 2 PCs as regressors, brown) methods are plotted. The two vertical black dash lines plot the onset and offset of stimuli, with a duration of 13.6 s. Responses of zero are relative to the average of 5-s baseline. (TIF) [file pone.0244186.s001.tif]

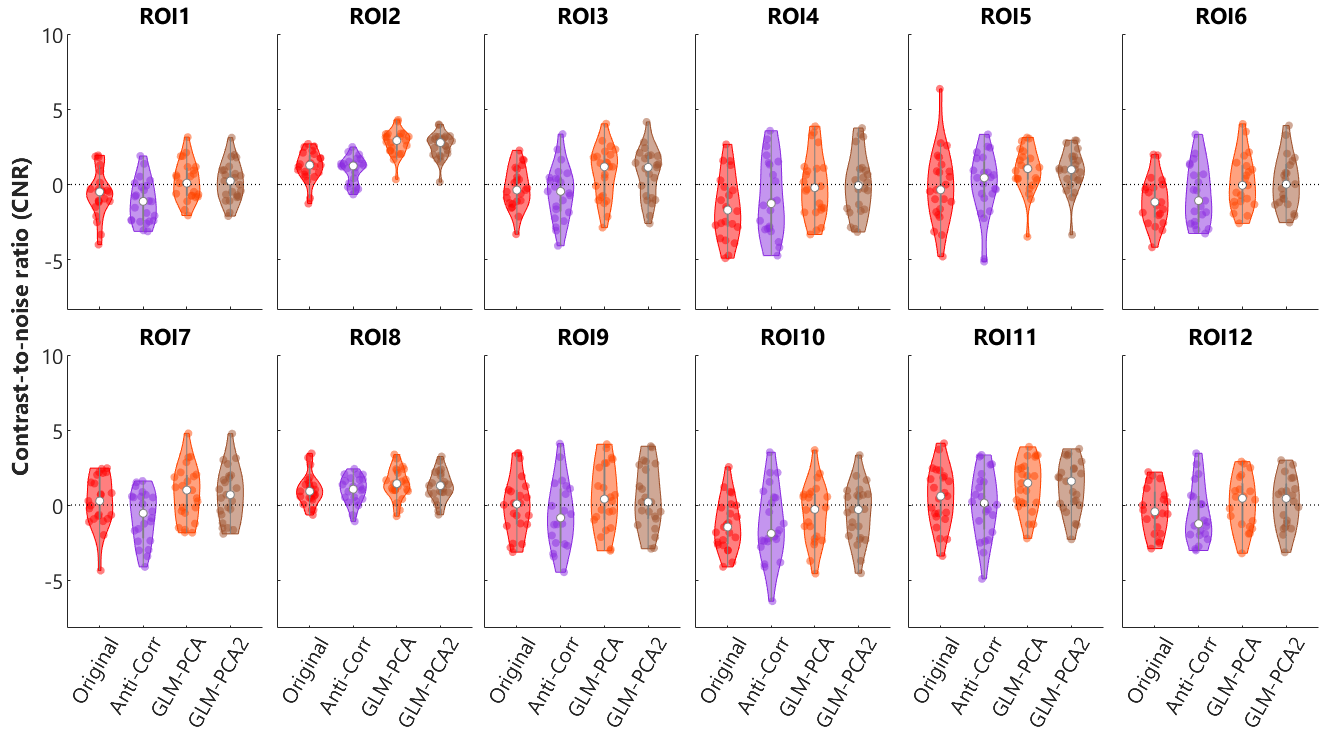

Supplement: S2 Fig — Results from channels with SCI> = 0.75 were included. Violin plots show the CNRs from HbO responses before (Original, red) and after applying Anti-Corr (purple) or GLM-PCA method (yellow), or GLM-PCA2 (brown) in individual participants in 12 regions of interest (ROIs). Results from the multilevel linear model analysis on the CNR values found a significant interaction between methods and ROIs (χ2(33) = 58.74, p = 0.004), and significant differences between methods (χ2(3) = 239.92, p < 0.001), and between ROIs (χ2(11) = 89.39, p < 0.001). Results from the Benjamini-Hochberg post-hoc tests revealed no significant differences in CNR values between Anti-Corr and the Original method (p = 0.26), but demonstrated significant differences between the two GLM-PCA methods and the Original (p < 0.001), between the two GLM-PCA and Anti-Corr (p < 0.001), but no differences between GLM-PCA and GLM-PCA2 (p = 0.77). (TIF) [file pone.0244186.s002.tif]
