# Supplementary material for: Comparing fNIRS signal qualities between approaches with and without short channels
Source: PLoS One. 2020 Dec 23;15(12):e0244186. doi: 10.1371/journal.pone.0244186 (PMC7757903; doi:10.1371/journal.pone.0244186)
Supplement: Attachment — Submitted filename: Reviews.pdf [file pone.0244186.s004.pdf]

The paper entitled "*comparing fNIRS signal qualities betrween approaches with and without short channel*" Is a well written paper that provides interesting information about methodological choices for the NIRS data processing. I found the paper easy to read and hypotheses and results are easy to understand. The final contribution would certainly be valuable for future functional NIRS studies. This being said I think the paper would greatly benefits from some clarifications of methodological choices and reorganization of some parts. You will find below a list of questions and recommendations related to it.

- **Introduction**

  - A description of the different systemic components (HR, Mayer waves…) and how they interfere with the neural signal could be useful for the naive or NIRS beginners. For instance how the HR could be summed on block averaging (e.g., Kirilina 2012) and how Mayer waves' frequency overlap with the neurovascular response.

  - Why did you choose to use GLM-PCA and what's the difference with other regression like the almost conventional Ac= AL-α.AS (Scholkmann 20014 and Nirx short-channel ressources)?

  - You choose PCA over ICA. Please justify this choice.

- **Method**

  - I would suggest justifying more the limitation of the study in the comparison of only two methods. Indeed, you mentioned 5 different technics in the introduction and, if the choice of the Gold standard short channel is obvious, the choice of Anti-Corr only is not justified enough.

  - I'm aware that fNIRS processing pipeline has not reached a consensus yet, however I have few concerns about your choices that you may need to clarify/justify.

  - Filtering: You did not mention (or I missed it) the first band-pass filter you applied on the ΔHb [0.1 1.5] Hz in the text, hence no justification for this range is available. Since there seem to be some agreement for a [0.01 0.09] frequency band (see Pinti et al., 2019), why did you choose these values?

  - Short-channel regression: You chose to apply the short-channel correction over the averaged concentration data whereas numerous researchers have stated that it is better to do it on the OD. Even the code provided by Nirx for Homer2 states that the regression should be applied on the OD. Could you justify this choice and state how this could affect the preprocessing results.

  - ROIs: A figure with the ROIs would really help visualizing your results and discussion. In addition a brief justification for this choice should be written in the Method section.

- Statistics: It’s a bit annoying to discover the statistical analyses in the results section. I would prefer an *a priori* description of them in the Method section.

- What was the device acquisition sampling frequency?

- Participants: Please detail the material used for hearing assessments.

L120. Normal channel distance average is certainly around 30 mm but according to the 10-20 positioning system you should have variations around this distance (it can be roughly from 25 up to 40 mm according to Nirsite). You need to add more information about this issue and how it can be partially corrected using Nirx spacers (only to prevent channel from exceeding 30mm).

L122. Please provide more information about cap positioning over the head to ensure homogeneity across participants.

L133. Define IAC the first time you use it.

L169. Justify such difference compared to Pollonini. If you have clearly described why you need to have at least 4 short channels of correct quality, it seems that a SCI threshold of 0.15 is very low. Looking at your figure 3, if you choose a criterion of SCI >0.5 for instance, you'll be closer to Pollonini's and will only lose participant N° 19 while still keeping an average of 6.9 short channels for the remaining 22 participants whit a better quality.

From L189 to L211: this part could be simplified and clearer if you state only once that all three data types (Not corrected, Anti-Corr and Short-channel) where finally filtered and block-averaged in the same way.

- **Results**

  - L353. As a reader I would be curious to have a look at the 2 principal components.

- **Discussion**

  - As APD are known to have a better light sensitivity, I’d like to know whether your conclusion would remain the same for normal/standard photodiode.

  - I’m a little bit surprised regarding the results for the auditory cortex. Since several studies have succeeded in probing this region with conventional NIRS setup (see references below), I’m not convinced about your argument. At this point, I recommend doing a Monte Carlo simulation of photon travel with your setup in AtlasViewer to discuss your result with actual depth penetration values. I’m currently doing research with a comparable montage and have quiet descent sensitivity in the superior temporal sulcus, precisely in the primary auditory cortex. Here again a picture of the ROIs would help in the results interpretation.

Ref:

Santosa, H., Hong, M. J., & Hong, K. S. (2014). Lateralization of music processing with noises in the auditory cortex: an fNIRS study. Frontiers in Behavioral Neuroscience, 8, 418.

Y. Minagawa-Kawai, K. Mori, I. Furuya, R. Hayashi, Y. Sato. Assessing cerebral representations of short and long vowel categories by NIRS. Neuroreport, 13 (2002), pp. 581-584

Plichta, M. M., Gerdes, A. B., Alpers, G. W., Harnisch, W., Brill, S., Wieser, M. J., & Fallgatter, A. J. (2011). Auditory cortex activation is modulated by emotion: a functional near-infrared spectroscopy (fNIRS) study. Neuroimage, 55(3), 1200-1207.

- **Figures**
  Please cite image sources (NIRX/Nisite for instance)
