# Supplementary material for: Comparing fNIRS signal qualities between approaches with and without short channels
Source: PLoS One. 2020 Dec 23;15(12):e0244186. doi: 10.1371/journal.pone.0244186 (PMC7757903; doi:10.1371/journal.pone.0244186)
Supplement: Attachment — Submitted filename: Response to Reviewers.docx [file pone.0244186.s005.docx]

Dear Editor,

We want to thank you and three reviewers for providing us with excellent feedback to improve the quality of this manuscript. We have worked to clarify any underlying confusion and addressed all of the comments. We provide a point-by-point response below, with responses to reviewer #1 are on pages 1-5, responses to reviewer #2 are on pages 6-11, and responses to reviewer #3 are on pages 12-14. We look forward to hearing back from you about the suitability of the paper for publication.

**Response to Reviewers**

Reviewer #1: The paper entitled “comparing fNIRS signal qualities between approaches with and without short channel” Is a well written paper that provides interesting information about methodological choices for the NIRS data processing. I found the paper easy to read and hypotheses and results are easy to understand. The final contribution would certainly be valuable for future functional NIRS studies. This being said I think the paper would greatly benefits from some clarifications of methodological choices and reorganization of some parts. You will find below a list of questions and recommendations related to it.
• Introduction
- A description of the different systemic components (HR, Mayer waves…) and how they interfere with the neural signal could be useful for the naive or NIRS beginners. For instance how the HR could be summed on block averaging (e.g., Kirilina 2012) and how Mayer waves’ frequency overlap with the neurovascular response.

***Response:*** *The authors reorganized and revised the Introduction. In the revised document, we described the different systemic components in the recorded fNIRS signals (lines 33-39) and the overlap between Mayer waves and fNIRS signals of interest in the frequency domain (lines 43-44).*

- Why did you choose to use GLM-PCA and what's the difference with other regression like the almost conventional Ac= AL-α.AS (Scholkmann 20014 and Nirx short-channel resources)?

***Response:*** *In this study, the conventional methods were not used, which directly subtract the short-channel responses from regular channels using a linear regression or adaptive regression, because these direct-subtraction methods assume that the local scalp responses measured by short channels also exist in the regular channels. Hence, these methods are suitable for studies that use dual optodes, i.e., by pairing one light source with one short-channel detector and one regular-channel detector, or in studies that short channels were located within 15-mm from regular channels. However, this was not the case in the current study that had a wide coverage of the montage with only 8 short channels. We elaborated on the choice of GLM-PCA method (lines 73-84) in the revised document.*

- You choose PCA over ICA. Please justify this choice.

***Responses:*** *We elaborated on the principles of using both PCA and ICA to reduce the noise and systemic responses in fNIRS signals and acknowledged the potential issues of using both ICA and PCA without shorter channels (lines 45-62). We also explained that this study chose PCA but not ICA, because a previous study by Virtanen et al (2009) compared the performance between ICA and PCA and found that ICA was inferior to PCA.*

• Method
- I would suggest justifying more the limitation of the study in the comparison of only two methods. Indeed, you mentioned 5 different technics in the introduction and, if the choice of the Gold standard short channel is obvious, the choice of Anti-Corr only is not justified enough.

***Responses:*** *The Anti-Corr method has been widely used in studies when short-channels were not available, and also due to its relatively high efficacy in reducing the systemic noise in fNIRS data (line 110-116). However, the fundamental assumption of this method was not entirely valid. Hence, the current study compared the efficacy in reducing systemic noise between methods with and without short-channels.*

- I’m aware that fNIRS processing pipeline has not reached a consensus yet, however I have few concerns about your choices that you may need to clarify/justify.
- Filtering: You did not mention (or I missed it) the first band-pass filter you applied on the ΔHb [0.1 1.5] Hz in the text, hence no justification for this range is available. Since there seem to be some agreement for a [0.01 0.09] frequency band (see Pinti et al., 2019), why did you choose these values?

***Responses:*** *The authors appreciate the reviewer pointing out the mistake that we made while plotting Figure 2. The bandpass filter that was used for GLM-PCA method was 0.01 – 1.5 Hz, but not 0.1 – 1.5 Hz. Before calculating the block-average, a (second) band-pass filter (0.01 – 0.1 Hz) was applied for all three methods, which was similar to the filter generally used. We corrected the mistake we made in figure 2 and explained about the band-pass filter in the text (lines 250-252) in the revised document. Also, we reanalyzed the data using the bandpass-filter 0.01-0.09Hz, instead of 0.01-0.1 Hz, and updated our results.*

- Short-channel regression: You chose to apply the short-channel correction over the averaged concentration data whereas numerous researchers have stated that it is better to do it on the OD. Even the code provided by Nirx for Homer2 states that the regression should be applied on the OD. Could you justify this choice and state how this could affect the preprocessing results.

***Response:*** *The study by Pinti et al. (2019) summarized the past research about pre-processing and filtering on fNIRS signal and found that in than 65% of research, the filtering (including spatial filtering, FIR, moving average) was conducted on the concentration data. Further, they compared the results from filtering on the concentration data versus filtering on the OD data and found no differences in the outcome. Hence, in the current study, the GLM-PCA was conducted on the calculated concentration data for each channel, rather than the OD data.*

- ROIs: A figure with the ROIs would really help visualizing your results and discussion. In addition a brief justification for this choice should be written in the Method section.

***Response:*** *The authors appreciate this suggestion. We showed the locations of channels above the cortex and how the channels were clustered into 12 in Figure 1D. In the methods section (lines 238-243), we described the details about ROIs.*

- Statistics: It’s a bit annoying to discover the statistical analyses in the results section. I would prefer an a priori description of them in the Method section.

***Response:*** *In the revised document, we had a separate subsection for Statistical analysis (lines 279-287). As we reanalyzed the data, instead of showing the correlations between HbO and HRF models, we calculated the contrast-to-noise ratios (CNRs, lines 272-277). We also re-did the statistical analysis on the CNR values from different methods, with 12 ROIs as dependent measures, and participants as a random variable.*

- What was the device acquisition sampling frequency?

***Response:*** *We appreciate this question, and in the revised document (line 182)*, w*e included that the sampling frequency for data collection in the current study was 3.91 Hz.*

- Participants: Please detail the material used for hearing assessments.

***Response:*** *In the revised manuscript (lines 139-141), we explained that a pure-tone audiometry via air conduction was performed to assess the hearing threshold of participants.*

L120. Normal channel distance average is certainly around 30 mm but according to the 10-20 positioning system you should have variations around this distance (it can be roughly from 25 up to 40 mm according to Nirsite). You need to add more information about this issue and how it can be partially corrected using Nirx spacers (only to prevent channel from exceeding 30mm).

***Response:*** *We appreciate this suggestion. In the revised manuscript (lines 164-168), we explained the positioning of optodes based on 10-10 system and the usage of plastic spacers to fix the channels distances to be no more than 30 mm.*

L122. Please provide more information about cap positioning over the head to ensure homogeneity across participants.

***Response:*** *In the revised document (lines 169-172), we explained in detail about the positioning of the cap, including centralizing the cap with Cz as the reference point, and using a chest strap for fixation.*

L133. Define IAC the first time you use it.

***Response:*** *We spelled out IAC as ‘Industrial Acoustics Company’ (line 170).*

L169. Justify such difference compared to Pollonini. If you have clearly described why you need to have at least 4 short channels of correct quality, it seems that a SCI threshold of 0.15 is very low. Looking at your figure 3, if you choose a criterion of SCI >0.5 for instance, you'll be closer to Pollonini's and will only lose participant N° 19 while still keeping an average of 6.9 short channels for the remaining 22 participants whit a better quality.

***Response:*** *The data presented in the current study were collected in three separate sessions, hence, the GLM-PCA method was conducted on the data from each session and then combined. Figure 3 shows the mean SCI for each short channel across three sessions of data collection. To clarify the confusion, we replotted figure 3 by showing the mean ± SEM SCI values of short channels (panel A) across three sessions for individual subjects. As shown in Figure 3A, in some participants, the same short channels had different SCI values in three sessions. Keeping SCI>=0.15 ensured that at least 4 short channels were involved in GLM-PCA for data collected from each session. Further (see lines 216-218), the short channels in the current study were located above the bilateral frontal and temporal cortex, aiming to sample a ‘global’ component for the montage. A threshold of SCI>=0.15 kept channels from both the frontal and temporal cortex being involved.*

From L189 to L211: this part could be simplified and clearer if you state only once that all three data types (Not corrected, Anti-Corr and Short-channel) where finally filtered and block-averaged in the same way.

***Response:*** *In the revised document (lines 232-271) we described the three methods separately, simplified the methods, but also elaborate on the GLM-PCA method to clarify the confusion.*

• Results
- L353. As a reader I would be curious to have a look at the 2 principal components.

***Response:*** *In the current study, the GLM-PCA was conducted on three sessions of data, separately, for each subject. The authors found it difficult to show results about the two PCs. Instead, in the revised manuscript (lines 256-258) we included the contributions of the two PCs to HbO and HbR data from short channels, 71.88%±7.65% and 58.66%±6.60%, respectively.*

• Discussion
- As APD are known to have a better light sensitivity, I’d like to know whether your conclusion would remain the same for normal/standard photodiode.

***Response:*** *In the current study, the regular channels were provided by the APD detectors, whereas the short-channel bundle was connected to an extra silicon photodiode detector (lines 157-158). This is a great point. Unfortunately, we were not able to argue here whether the short-channel method would be better if the short-channel bundle was connected to an APD detector. We hope to investigate this in the lab for future studies.*

- I’m a little bit surprised regarding the results for the auditory cortex. Since several studies have succeeded in probing this region with conventional NIRS setup (see references below), I’m not convinced about your argument. At this point, I recommend doing a Monte Carlo simulation of photon travel with your setup in AtlasViewer to discuss your result with actual depth penetration values. I’m currently doing research with a comparable montage and have quiet descent sensitivity in the superior temporal sulcus, precisely in the primary auditory cortex. Here again a picture of the ROIs would help in the results interpretation.

***Response:*** *In the revised document (Figure 1D), we showed the clusters of channels into ROIs and the locations above the cortex. In the revision (lines 380-389), we argue the suppressive responses in the ROIs above the temporal cortex could be due to two reasons. First, some channels in ROI6 (one the left), and ROI10 (on the right) were located around or above the auricles, where there was generally a lot of hair that might have contaminated the data. Second, some of the channels were located above the temporal sulcus, the channels might have limited access to the neuronal activity. Hence, we might not be able to record good signals from these ROIs.*

Refs:
Santosa, H., Hong, M. J., & Hong, K. S. (2014). Lateralization of music processing with noises in the auditory cortex: an fNIRS study. Frontiers in Behavioral Neuroscience, 8, 418.
Y. Minagawa-Kawai, K. Mori, I. Furuya, R. Hayashi, Y. Sato. Assessing cerebral representations of short and long vowel categories by NIRS. Neuroreport, 13 (2002), pp. 581-584
Plichta, M. M., Gerdes, A. B., Alpers, G. W., Harnisch, W., Brill, S., Wieser, M. J., & Fallgatter, A. J. (2011). Auditory cortex activation is modulated by emotion: a functional near-infrared spectroscopy (fNIRS) study. Neuroimage, 55(3), 1200-1207.

• Figures
Please cite image sources (NIRX/Nisite for instance)

***Response:*** *We added the citation in the caption for Figure 1.*

Reviewer #2: Overall, I think that this paper still lacks strong and clear results.
The manuscript requires some additional work to better present the results and, possibly, also the data analysis.
I have two main concerns about this paper.

[1] The first is methodological.
The GLM-PCA method includes a GLM model that uses as regressors, in addition to the two principal components of the PCA on the short channels, also information about the temporal locations of the stimuli, i.e. the “stimulus-regressors”.
So the GLM is “informed” that there are the stimuli.

After, the comparison between the three pipelines is performed on the capacity of recovering the effect of the stimuli.
I think that this is not completely fair, as the results from the pipeline with short-channels come from a step that uses the same information that is “searched” to process the signals.

- What would be the result of using a GLM only based on the two main components?
- Or, conversely, what would be the results on the two pipelines not based on short-channels when a GLM with “stimulus-regressors” is used?

***Response:*** *This is a great question. As the regular (30-mm) fNIRS channels recorded the event-related and non-related extracerebral responses and neuronal activity in the deeper tissue, we used the event-related regressors hoping to better represent the signals in the regular channels. In the revision, we reanalyzed the data by including a second GLM-PCA method, i.e., GLM-PCA2 with only the two PCs as the regressors for GLM (lines 266-270). We also calculated the contrast-to-noise ratios (CNRs) to evaluate the efficacy between methods in reducing the systemic responses in fNIRS signals (lines 272-277). Results from the two GLM-PCA methods are shown in Figure 5 and Figure 6 in the revised document. Statistical analyses found that both GLM-PCA (with event-related regressors and PCs) and GLM-PCA2 (only PCs) improved the signal qualities, but no significant differences in the CNRs values between the two GLM-PCA methods (p=0.992, lines 338-341). These results suggest that the PCs were not correlated with the event-related regressors, hence the event-related systemic responses do not dominate in the extracerebral tissues.*

[2] The second pertains to the organization of the study.
This is a methodological paper with the aim of comparing signal processing pipelines.
However, there is not a strong result on the comparison here.
Instead, there are many qualitative considerations (many figures, maybe too many) but not a clear quantitative assessment.

***Response:*** *We appreciate this comment. In the introduction (119-123), we elaborated on the reasons for showing the results about short channels (Figures 3, 4), that there is a paucity of information about the functioning of short channels and the data qualities that have been measured from individuals. We did take this suggestion and deleted one figure (Figure 5 in the original manuscript) that was no longer very informative.*

I think this is partially due to the organization of the manuscript.
First, the Methods section completely lacks any information about how the pipelines will be compared, and on what basis the comparison methods have been defined. These methods are mentioned in the Results but without any strong rationale (see also below).

Second, the Results section presents many findings that are not strictly “results”. They are more qualitative descriptions that do not add any real contribution to the study. I think the Results should focus only on the comparison of the pipelines, which is the aim of the manuscript.

Partially, it is also due to the fact that the comparison methods seem quite arbitrary: there is not a motivation about how and why the Authors decided to use the correlation with the HRF. Is this choice supported by some functional hypothesis? Are there other papers that adopt the same methods?

***Response:*** *We appreciate these comments. In the revised document (lines 272-277), we reanalyzed the data and calculated the contrast-to-noise ratios (CNRs) from HbO response, which has been widely used to reveal the signal quality in fNIRS and fMRI studies, with larger CNRs suggesting better signal-to-noise ratios.*

*Further, we included a subsection of statistical analysis (lines 279-287), and described how we evaluated the efficacy from different methods by examing the CNRs between different methods.*

On one side, I understand that it is hard to objectively compare the results of the three pipelines, having not a ground truth about what an ideal signal would look like.
However, the methods adopted rely on many - explicit or implicit - assumptions and so they appear quite arbitrary. For instance: why using the ROI? Is it correct to expect the same (temporally and spatially) brain activity response on all the ROIs? Are all ROIs involved in the processing of the stimulus? What makes the correlation with the expected HRF an appropriate quality measure?

***Response:*** *In the revised document (238-244), we explained that the channels were clustered into ROIs based on their anatomical positions, i.e., some channels overlapped and some were adjacent. As the ROIs were located above the language processing pathways, from the bilateral frontal to temporal cortex (Figure 1 C-D), and fNIRS data were collected when participants were listening to sentences in noise, we did anticipate responses in all the ROIs. We then conducted the statistical analyses based on the CNRs for HbO responses from all the ROIs.*

Finally, it is partially because it is not completely clear what is the object of the evaluation.
Is it the use of short-channels, or the GLM-PCA algorithm? Or both?
If the short-channels: what about using other preprocessing algorithms?
If the algorithm: what about using the GLM-PCA on the “usual” channels?
If both: why both? And why was the GLM-PCA chosen?

- The Authors should try to better organize the manuscript and adequately motivate the methods adopted.

***Response:*** *We revised and rewrote the introduction section to clarify the purpose of the study, i.e., comparing the efficacy of the GLM-PCA method by including short channels for reducing the systemic responses, with the widely used Anti-Corr method. In the introduction, we also elaborated on the shortness of using GLM-PCA or ICA methods without short channels (lines 56-62). When systemic responses were related to the neuronal responses, i.e., event-related, PCA and ICA methods based on the regular channels would fail to separate them hence not able to regress the systemic responses out.*

*We also explained the direct subtraction methods by using a linear or adaptive regression to subtract the short channel signal directly from regular channels (lines 75-84). These methods were more suitable for regular and short channels that were connected through the same light source (dual optodes) or when they were within 15-mm distance, hence measuring the same local extracerebral responses. However, these methods were not suitable for the study that had a larger montage but only 8 short channels.*

- The purpose of the study should be better identified and the Results section should focus only on the presentation of the quantitative results.

***Response:*** *We appreciate this comment. As explained above, we reworded the introduction to better identify the purpose of the study. In the results section, we only showed the block-averaged responses in the short channels (Figure 4), in the ROIs for different methods (Figure 5), and the quantitative results about CNRs to (Figure 6). We revised the text, correspondingly.*

Then there are some major and minor issues.

Major
- The preprocessing procedure involved a step in which some channels were removed. As this step operates on each subject independently, it is important to state how many channels have been used in the downstream analysis (see Bizzego et al. 2020);

***Response:*** *The authors appreciate this recommendation.* *In the revised document (Figure 3B), we showed the number of short and regular channels that were involved in each dataset per participant. We also reported (lines 294-297) the mean ± SD number of short and regular channels that were excluded, separately.*

- Short channels are used to different sources of noise from the standard channel, as mentioned in the Introduction. However, in this study the Authors adopt a number of countermeasures to avoid and remove noise: they ask the subjects to remain still and they filter the signals to remove noise. The Authors should comment on that, as the adopted countermeasures could make useless the use of short-channels and other processing procedures.

***Response:*** *The short channels were adopted to reduce the extracerebral responses, including task-evoked and non-evoked systemic responses, which contributed a lot to the signals recorded in the regular channels. However, unlike motion artifacts that could be reduced using wavelet analysis or PCA method (without short channel), the systemic responses that overlap with neuronal signals in the frequency domain are difficult to remove. Further, the reason we asked participants not to make dramatic movements was too avoid optodes shifting on the head during data collection, which could cause poor data quality.*

- Why only the first two principal components have been used? Why two? This choice should be better motivated.

***Response:*** *Two PCs were chosen to represent the event-related and non-related systemic responses in the extracerebral tissue (lines 125-126).*

- Figure 2: it seems that the standard channels in the short-channel “track” undergo two bandpass filters: the first with 0.1 - 1.5 Hz, and the second 0.01-0.1 Hz. But this is not mentioned in the manuscript. Was the first filter really applied? If so, then the two filters would actually filter al the frequency components, as the bands do not overlap.

***Response:*** *We apologize for the typo in Figure 2. The filter that was applied before the GLM-PCA method was 0.01-1.5 Hz, to* *remove the low and high-frequency physical noise. We corrected the mistake we made in figure 2 and explained about the band-pass filter in the text (lines 250-252) in the revised document.*

- Why were the channels merged into ROIs? How were these ROIs created? Do they group channels according to the Brodmann Areas? How many channels have been used for each ROI and participant?

***Response:*** *The channels were clustered into ROIs based on the anatomical positions. Except ROI2 and ROI8 that consisted of 2 adjacent channels, all ROI consisted of 4 channels (two overlapping channels and two adjacent channels), We showed the locations of the channels above the cortex, and the number of channels and how they were clustered into ROIs in Figure 1D.* Correspondingly, in the methods section (lines 238-243) we described the ROIs in detail.

- L. 291-293: why the multilevel linear model? What is the hypothesis that is tested? Any reference from the literature? This sentence is also unclear.

***Response:*** *In the revised document (lines 279-287) we added the subsection of statistical analysis to test the efficacy of the Anti-Corr and GLM-PCA methods in improving the signal quality. The multilevel linear model was conducted on results from all subjects in 12 ROIs for different methods to examine whether there were any significant differences in signal quality between no-correction and after correcting systemic response. The multilevel linear model was used, instead of a repeated-measure ANOVA (RM-ANOVA) because data collected from 12 ROIs were dependent, whereas RM-ANOVA requires the different ‘measures’ were independent. We also included the citation of Bliese 2000.*

*Bliese, P. D. (2000). Within-group agreement, non-independence, and reliability: Implications for data aggregation and analysis.*

- L. 298-300: what is this follow up contrast analysis? Contrast between what? If reported then it should be described and motivated in the Methods section.

***Response:*** *We re-did the statistical analysis, as described above (in the subsection of Statistical analysis, lines 279-287). A post-hoc test was conducted with Benjamini-Hochberg for multiple comparison correction, to examine whether there were significant differences in the CNR values from different values.*

Minor
- L. 94: I think that what is compared should be the efficacy of the pipelines and not the “quality” of the signals. The quality of the signal is something that is given, the pipelines can be more or less robust to low quality signals.

***Response:*** *We agree with this comment and changed the use of ‘quality’ to efficacy in the introduction (line 117) and through the text.*

- L. 119-120: “A single source …” This sentence is not clear

***Response:*** *We reworded this sentence (lines 152-153) as ‘A light source connected to detectors located at about 30-mm distance provided fNIRS channels that collected signals.’*

- L. 134-135: I think “optimization” is not an appropriate term. Optimization usually indicates a procedure where the parameters of an algorithm are tuned to maximise the efficacy.

***Response:*** *We reworded the sentence (lines 175-176) as ‘*the gains of light intensity at the APD detectors were checked to ensure that all the channels had at least ‘acceptable’ light intensity’ *to avoid using the term ‘optimization’.*

- L. 156-162: the description of this step could be more accurate. Maybe “derivative” is more appropriate than “deviation”; I think the Authors also used the absolute values of Xi

***Response:*** *We changed the ‘deviation’ to ‘derivative’ (line 200), and revised the formula to be ‘X_i_* > mean(abs(*X*)) + 2*std(*X*)’ *(lines 201-202).*

- L. 171-173: This sentence should motivate the use of ‘4’ as the minimum number of short-channels. But it is quite unclear. What do the Authors mean by ‘low cost budget”? Why is budget important here?

***Response:*** *Quite often, it is not technically practical to include many short channels, hence with a limited number of short-channels that could provide robust and good performance in reducing the systemic response is important.*

The following comments are on parts of the text that I think could be removed. In case the Authors decide to put them somewhere else (e.g. Supplementary Information)
- L. 219-221: How the Authors verified this? I think this should be presented as an hypothetical explanation.
- L. 225-228: This is not a finding as the threshold was specifically set to obtain these results

***Response:*** *In the results section (290-299), we deleted the descriptive results about the short and regular channels, instead, we only kept the stats about how many short and regular channels were included for further analysis.*

- L. 237-245: the Methods section does not mention the group-averages on the short-channels; this part comes from nowhere. Why is the readed introduced to these results? What is the purpose of showing them? If reported, they should be better motivated and logically included in the procedure.

***Response:*** *In the introduction (lines 119-123) we explained that despite the popularity of including short channels in fNIRS studies, it is unclear how the short-channel responses look like, and how they work in individuals subjects Hence, the current study provided the details for references in future studies. Subsequently, we included in the analysis of block-averaged responses in short channels (lines 233-234).*

References
Bizzego et al. (2020). Commentary: Current status and issues regarding pre-processing of fNIRS neuroimaging data: an investigation of diverse signal filtering methods within a general linear model framework. Frontiers in Human Neuroscience
doi: 10.3389/fnhum.2020.00247

Reviewer #3: This paper is concerned with a comparison between two methods of removing systemic influence from the fNIRS signal. The authors have compared the anti-correlation approach with a PCA method based on short channels. This is a useful paper for researchers working with fNIRS, especially as some aspects of signal treatment and fNIRS quantification remain in development.

In this particular paper, 23 participants listened to sentences as the primary experimental task. There is no description of the sentences/stimulus material. I can see how long each lasted for and the temporal characteristics of stimulus presentation, but I would like some concrete examples of the material and confirmation that no motor response was required from the participant.

***Response:*** *In the revised document (lines 143-144) the sentence/stimulus structure was described, with an example sentence ‘Bob has six green hats’. During the experiment (lines 188-190), participants were required to listen to blocks 5 sentences and count the number of color words in each block and responded by pushing mouse buttons to indicate whether it was an even or odd number. In this way, dramatic or frequent motor responses were avoided.*

Figure 2 provided a clear presentation of the different treatments of the fNIRS data that form the basis for this paper. However, there was one aspect that I did not understand and I'm concerned that it may confound the comparison that the authors wish to make. The original data and anti-correlation data are subjected to the same signal processing as the GLM_PCA route except for a bandpass filter of 0.1-1.5Hz that was only applied to the GLM-PCA paper. What was the purpose of this filter? Was it only applied to the 8mm (short channels)? It was not clear to me from reading the paper, why this filter had been applied to that particular data set and not the other two.

***Response:*** *We apologize for the typo we made in Figure 2. The filter that was applied before GLM-PCA method was 0.01-1.5 Hz, on both the short and regular channels, to* *remove the low and high-frequency physical noise. We corrected the mistake we made in figure 2 and explained about the band-pass filter in the text (lines 250-252) in the revised document.*

The authors are very diligent with respect to filtering the signal quality utilising the SCI method developed by Pollonini et al and showing the results in Figure 3. On page 10, they state that the original method used <0.75 as an exclusion criterion, which they felt was too strict, so instead they opt for a cut-off frequency of 0.15, which is even lower. This part of the text does not make sense and should be corrected, in addition, the authors should justify the basis of their adjustment to the original exclusion criterion - not simply say that it was too strict, i.e. if they had used it, how much data would have been lost?

***Response:*** *The main reason for keeping* SCI>=0.15 was to keep at least 4 short channels for PCA analysis. We elaborated on the reason (lines 210-218) as “In the current study, the 8-mm short channels had relatively poor success in quite a few subjects (Fig. 3A) likely due to hair artifacts. Hence, a lower cut-off frequency (SCI >= 0.15) was set compared to that in Ref [35] to ensure for each participant there were at least 4 shorter channels remaining for further analysis of the data from each session.” As shown in Figure 3A, with an SCI>=0.75 would not be able to do that. Further, keeping SCI>=0.15 ensured that short channels from both the frontal and temporal lobes were included in the PCA, to better represent the extracerebral components across a wide brain region.

I would also like more details of the PCA described on page 11. My reading of the text suggests that the PCA is based on all short-channel data, or was the PCA specific to location or hemisphere? This was not clear to me. I also did not quite understand how regressors were calculated, did the PCA represent HbO or Hbb or the HRF function? Finally, the authors extracted the largest two PCs as regressors for the GLM, but I would like to know what proportion of the total variance was accounted for by these 2 PCs.

***Response:*** *We appreciate this feedback. We included more details about the PCA and explained that PCA was performed on the HbO and HbR responses, separately, from all short channels (out of eight) with SCI >=0.15. In lines 255-257, we also showed the contribution of the two PCs to the variances of HbO (*71.88% ± 7.65%*) and HbR data (*58.66% ± 6.60%*), separately. We also clarified that the GLM-PCA methods were also conducted on the HbO and HbR data, separately, for each regular channel from individual participants (267-270).*

I was also very unclear about how the ROIs had been derived from the montage and the text on this topic at the bottom of page 13 should be expanded to make this clear. Perhaps the authors could indicate the ROI on the montage diagram shown in Figure 1?

***Response:*** *We showed the locations of the channels above the cortex, and the number of channels and how they were clustered into ROIs in Figure 1D.* Correspondingly, in the methods section (lines 238-243) we described the ROIs in detail.

When it comes to the comparison between the three data sets, I felt that the authors relied too heavily on data visualisation. The authors make the claim that GLM-PCA method reduced the suppressive component and we can see that in the plots, or at least we can for HbO, the deoxygenated signal is more difficult to see. Their method of testing correlation with HRF is OK but a little indirect. Other authors, e.g. Fairclough et al (2018) who have compared anti-correlation methods with raw data have done by comparing effect sizes in response to independent variables across different ANOVA. I would prefer the authors to adopt a similar approach by comparing activation during pre-stimulus period with activation during the presentation of stimuli via ANOVA. If the authors are concerned about sphericity, they can test each data set individually. I feel that this is important because most researchers want to know if these methods will make their data more sensitive to experimental effects - and this aspect is not really addressed in the current manuscript.

***Response:*** *We appreciate this feedback and reanalyzed the data by calculating the contrast-to-noise ratios (CNRs) to reveal the signal quality from each method (lines 272-277). We showed the CNR values in Figure 6. We re-did the statistical analysis (lines 279-287), by using a multilevel linear model to examine the differences between the CNR values from different methods, with ROIs as dependent variables, subjects as random subjects.*

As a final point, I would like the authors to consider in the Discussion whether their results would generalise to more complex tasks that involve more head movement and a motor component. They use a task that is very controlled (no response, minimal head movement) and they should consider whether their results would generalise to different types of task.

***Response:*** *We appreciate this comment. In the revised document (lines 423-430), we included that the short channel qualities from some participants varied across testing runs (Figure 3A) which could be due to the shifts of optodes on the head likely due to dramatic head movement, though participants were instructed not to do that. In the more complicated tasks that involved a lot of movements, it is important to fix the optodes on the head. Otherwise, the short channels would only end up measuring motion artifacts, hence not very helpful in reducing the extracerebral components in the regular channels.*
