# Supplementary material for: Comparing fNIRS signal qualities between approaches with and without short channels
Source: PLoS One. 2020 Dec 23;15(12):e0244186. doi: 10.1371/journal.pone.0244186 (PMC7757903; doi:10.1371/journal.pone.0244186)
Supplement: Attachment — Submitted filename: Response to reviewers.docx [file pone.0244186.s006.docx]

**Response to the editor and reviewers**

Dear editor,

We appreciate your feedback and the comments from all the reviewers on our last revision of the manuscript. We had revised the manuscript according to the comments from Reviewer #2 and provide a point-by-point response below. We look forward to hearing back from you about the suitability of the paper for publication.

Comments from Reviewer #2

I still have some methodological concerns:
- The authors should comment on an implicit assumption that I think it is critical: that an increase of HbO is expected during the stimulation. The CNR measure relies heavily on this assumption and would be pointless if no response is observed. The Authors should provide some references in the literature to support this assumption.

***Response:*** *We appreciate this comment. As the montage of the current study covered the ventrallater speech pathways, and fNIRS data were collected when individuals were listening to and processing sentences, we expected increased neuronal activity in all ROIs. In the revision (lines 149-152), we added the sentence with citation as ‘These ROIs covered the ventral-lateral pathways of cortical regions for auditory speech processing, including the frontal cortex, inferior prefrontal, anterior temporal cortex, and the superior temporal areas (see reviews with meta-analyses in [45, 46]).’*

*After describing the CNR values, we also added two sentences (lines 286-290) as ‘We expected greater than zero CNRs in all ROIs, which covered the ventral pathways of the auditory speech processing [45, 46]. When individuals were listening to and processing sentences, neuronal activity from cerebral tissues in the ventral speech pathway would increase, which was associated with increased DHbO responses through neurovascular coupling [54].’*

In parallel, instead of using custom ROIs, I would find more reasonable to group channels based on BAs, showing that those associated to the processing of the acoustic stimuli show greater activation.
- In any case, I urge the Authors to describe the rules they followed to compose the ROIs.

***Response:*** *We appreciate this comment. In the revision (lines 147-154), we explained how channels were clustered into the ROIs as ‘In total, forty-four 30-mm (regular) channels were included and grouped into twelve regions of interest (ROIs) based on their anatomical positions, with ROI1 – ROI6 on the left and ROI7 – ROI12 on the right side (Fig. 1C). These ROIs covered the ventral-lateral pathways of cortical regions for auditory speech processing, including the frontal cortex, inferior prefrontal, anterior temporal cortex, and the superior temporal areas (see reviews with meta-analyses in [45, 46]). As shown in Fig. 1C, each ROI consisted of 4 adjacent or crossing channels (labeled circles), which were aimed to measure signals from neighboring local brain regions. Except that ROI2 and ROI6 only consisted of two crossing channels.’*

*We apologize that it is difficult to cluster channels into ROIs based on Brodmann areas (BAs) for two reasons. First, some of the 30-mm channels might cross a few BAs. Second, we used the plastic spacers to ‘reposition’ the optodes when the distances for the channels were farther than 30-mm on the 10-10 system. The repositioning helped secure the fNIRS physical data quality, but might cause the optodes to be shifted from their positions on the 10-10 system. However, as mentioned above, the montage of the current study covered the ventral-lateral pathways for auditory speech processing, we expected all these regions were involved (whether in similar or different ways).*

The major issue is still about the organization of the contents in the manuscript and readability of the text.

- description of methods in the introduction: I suggest to first describe all methods that do not require short channels, and then those with short channels.

***Response:*** *We appreciate this suggestion! We have moved the paragraph in lines 105-116 (in the last version) that described the Anti-Corr method without short channels earlier so that it appears before we discuss short-channel methods. Please see lines 64-74 in the revised manuscript.*

- L98-104 interrupt the logical flow of the description and they raise a point that is never addressed later. Maybe this paragraph can be removed.

***Response:*** *We appreciate this feedback and considered the suggestion. We decided to keep this paragraph as it can be helpful for readers who want to know how short the distances of the short channels can be, and why they cannot be shorter than 8 mm, which is the length used in the current study? To improve the logical flow of the text, we rephrased the content in lines 117-121.*

- L117-133 this part is very hard to follow, some sentences are also ambiguous or too complicated. I strongly suggest to rephrase and reorganize the structure of this paragraph. I understand that the qualitative description of the brain response in short channels is considered of potential interest for the reader, but it is important to give some structure to this part: try to distinguish the different aims and between qualitative and quantitative results. I would suggest to keep the term "data" only to indicate signals or experimental measures. Here the authors probably mean "information"/"description" of the functioning of the short channels.

***Response:*** *We appreciate these comments. In the revision (lines 119-132), we started with our first aim of this study, i.e., to provide detailed information of the short-channel functioning. Then we described the fact that two GLM-PCA methods with short channels were employed, the performance of which in reducing systemic responses was compared with that from the Anti-Corr method. We evaluated the performance by calculating the contrast-to-noise ratios, followed by our predictions.*

- L53 and throughout the text: please ensure that the "Ref [x]" is an acceptable format to cite references;

***Response:*** *We fixed this through the text.*

- The description of the stimuli should state how many different stimuli are used, if they were repeated etc. I feel that the complete description of the experimental settings is needed to understand how the data were collected. Some of these details are reported after in the text, but I think it would be clearer to aggregate these details here. This should also be the place where the report what are the different experimental conditions and, in particular, why the chose this, and why only one. - L182-190: as previously commented, I would avoid separating the description of the experimental design from the description of the stimuli. Also the description should be complete and authors should motivate why the only focused on one condition.

***Response:*** *We appreciate these comments. In the revision (lines 180-198), we put stimuli and experimental design together and elaborated on the experimental conditions. The data presented in this manuscript were part of a larger study to examine the listening effort in eight different hearing conditions. In footnote 1, we explained that the aim of the current study was to examine the efficacy of using short-channels rather than listening effort, hence we only presented data from one of the easiest hearing conditions.*

- L152-153 The rephrased sentence is still unclear: maybe the authors just wanted to define what is considered to be a "channel" in fNIRS data? If so, also take the opportunity to clarify other points: how many total "regular" channels have been collected for each signal (should be 40: this information can be extrapolated but it is better to explicitly state that); what do they mean with "overlapping" channels: I think (hope) this does not mean that they refer to the same anatomical location (i.e.: same detector-source pair), so probably the Authors mean "near" instead of overlapping.

***Response:*** *In the revision (line 147), we included the sentence ‘In total, forty-four 30-mm (regular) channels were included.’ We agree that the term ‘overlapping’ channels is not accurate. We meant channels between different source-detector pairs that cross in the middle (so the mid location is identical in the two channels, although the optodes are oriented differently). In the revision, we used the term ‘crossing’ channels, instead.*

– I find the description of the montage (L157-168) a bit rough: what is a "bundle" in this case? please avoid ambiguous terms. Figure 1A is not very informative, it can be removed; channels in Figure 1C should be highlighted; Figure 1C and 1D can be merged if different colors are used to highlight channels belonging to different ROIs. Credits of Figure 1B are missing - I think. L166-168: I think that what authors mean is that whenever the detector was placed more than 30 mm from the source, the detector is re-positioned at 30 mm from the source, by means of a custom plastic holder.

***Response:*** *We appreciate all the suggestions! In the revision (lines 161-163), we rephrased the sentence about short channels as ‘Eight 8-mm channels were also used, whereby an extra silicon photodiode detector was split into 8 groups of dual-detectors (Fig. 1A, green arrows), with each group surrounding a light source at 8-mm distance. (Fig. 1C green dots and green lines). In lines 158-159, we rephrased the description of the plastic spacers as they ‘reposition the two optodes and keep the distance between them as 30 mm.*

*We replotted Figure 1, by merging Figure 1C and 1D and highlighted/colored the channels. We kept Figure 1 A as it shows how the short channels were connected. Please see the revised Figure 1. In the caption for Figure 1, we added more explanations about panel B as ‘Pairing a light source (in red) and a regular detector (in blue) at 30 mm distance provides a regular fNIRS channel, which measures response from the local region between them, including from the shallower extracerebral tissue and deeper cerebral tissue. Pairing a short channel detector (in green) at 8 mm distance from the light source provides a short channel, which measures signals only from the extracerebral tissue.’*

- Please use mathematical formulae and symbols; first write the equation, then describe the terms in the equation.

***Response:*** *We simplified the language and removed the ‘formula’ as it became unnecessary. Please see lines 208-211.*

- L205: "0.5 - 1.5 Hz signals" does not mean anything. What do the authors mean? Are these the original signals after the 0.5-1.5 Hz bandpass filtering?

**Response:** *We added the sentence (see lines 212-214) “A third-order Butterworth bandpass filter with cut-off frequencies of 0.5 and 1.5 Hz was applied to raw fNIRS intensity data to extract heartbeat signals in individual channels.”*

- Setting such a low SCI is quite critical. I would prefer to see the analysis on fewer subjects than a pipeline with such extreme parameter values. I would suggest to include a supplementary material with the results with the original SCI=0.75 threshold.

***Response:*** *We appreciate this comment. We provided block-averaged results and CNR values with a cut-off of SCI=0.75 in supplementary materials. Results from the statistical analyses demonstrated that the two GLM-PCA methods significantly improved the CNR values compared to the original signals and compared to the Anti-Corr method, similar to the results shown in the manuscript. Please see S1 Figure and S2 Figure, respectively. We also explained this result in the footnote 3 (line 354)*

- The pipeline description can be improved. The authors should separately describe the steps that are applied to all methods (1-5; 6 and 9) and those that are particular of each method (7 / 8 / 9). It is hard for the reader to exactly understand what was done.

***Response:*** *We reorganized Figure 2 and colored steps that were involved in each of the three methods differently.*

- The authors should comment on the fact that a decrease in HbO is observed in short channels. Was it expected? Why? Any similar finding in the literature.

***Response:*** *This is a great question. In the discussion section (lines 371-390), we discussed the ‘suppressive’ signals we observed in the current study, from both the short channels and regular channels. Similar results have been reported in the study by an fMRI-fNIRS study by Kirilina et al. 2012. Kirilina et al. proposed that the ‘suppressive’ responses might be due to vasoconstriction in the superficial skin tissue, which might occur when participants were under mental stress or performing arithmetic tasks, resulting in a decreased blood flow, thus decreased ΔHbO responses.*

- Figure 3B is not optimal to explain how many regular and short channels were actually used: I would try with an histogram.

***Response:*** *We replotted Figure 3 B by showing the histograms of numbers of short and regular channels in all the participants.*
